# Diagnostic Insights from Plethysmographic Alveolar Pressure Assessed during Spontaneous Breathing in COPD Patients

**DOI:** 10.3390/diagnostics11060918

**Published:** 2021-05-21

**Authors:** Camilla Zilianti, Pierachille Santus, Matteo Pecchiari, Edgardo D’Angelo, Dejan Radovanovic

**Affiliations:** 1 Dipartimento di Fisiopatologia e dei Trapianti, Università degli Studi di Milano, 20122 Milano, Italyedgardo.dangelo@unimi.it (E.D.); 2 Division of Respiratory Diseases, Dipartimento di Scienze Biomediche e Cliniche “L. Sacco”, Università degli Studi di Milano, 20157 Milano, Italy; pierachille.santus@unimi.it (P.S.); dejan.radovanovic@asst-fbf-sacco.it (D.R.)

**Keywords:** plethysmographic loops, PEEPi, expiratory flow-limitation, chronic obstructive pulmonary disease, respiratory function tests

## Abstract

Since its introduction in the clinical practice, body plethysmography has assisted pneumologists in the diagnosis of respiratory diseases and patients’ follow-up, by providing easy assessment of absolute lung volumes and airway resistance. In the last decade, emerging evidence suggested that estimation of alveolar pressure by electronically-compensated plethysmographs may contain information concerning the mechanics of the respiratory system which goes beyond those provided by the simple value of airway resistance or conductance. Indeed, the systematic study of expiratory alveolar pressure-flow loops produced during spontaneous breathing at rest has shown that the marked expansion of expiratory loops in chronic obstructive pulmonary disease patients mainly reflects the presence of tidal expiratory flow-limitation. The presence of this phenomenon can be accurately predicted on the basis of loop-derived parameters. Finally, we present results suggesting that plethysmographic alveolar pressure may be used to estimate non-invasively intrinsic positive end-expiratory pressure (PEEPi) in spontaneously breathing patients, a task which previously could be only accomplished by introducing a balloon-tipped catheter in the esophagus.

## 1. Introduction

Since its introduction in the clinical practice, body plethysmography proved a valuable tool in the diagnosis of respiratory diseases, thanks to its ability to provide a non-invasive estimation of absolute lung volumes [1] and airway resistance (R_aw_) [2,3,4]. A complete discussion of the technical and clinical aspects of the plethysmographic technique can be found in excellent reviews previously published [5,6,7] and is beyond the scope of the present work. The present review will focus on the possible pathophysiological and clinical insights obtainable from the assessment of alveolar pressure (P_alv_) with a body plethysmograph in patients with chronic obstructive pulmonary disease (COPD).

Knowledge of P_alv_ is an implicit requirement for R_aw_ estimation, as the latter parameter is the ratio between the driving pressure, that is airway opening pressure minus P_alv_, and the flow. With a plethysmograph, the changes of P_alv_ are measured in terms of changes in lung volume due only to compression or decompression of the gas inside the lung (ΔV_M_). If, at a given instant, the total volume of gas in the respiratory system (*V_rs,t_*) is known, Δ*V_M,t_* can be converted to *P_alv,t_* using the following equation:

(1) $$P_{alv,t}=\frac{P_{B}\Delta V_{M,t}}{V_{rs,t}+\Delta V_{M,t}},$$

where *P_B_* is barometric pressure minus water vapor pressure (note that, in the equation, *P_alv,t_* is referenced to *P_B_*). The challenging technical problem of this measurement is that, during the act of breathing, the changes of gas volume of the respiratory system independent of mass flow (called shift volume, ΔV_S_) are not due solely to ΔV_M_. In fact, ΔV_S_ is also influenced by thermal phenomena (ΔV_th_), as inhaled air is warmed and humidified in the respiratory system, and exhaled air is cooled and deprived of water vapor [2], and by metabolic phenomena (ΔV_met_), as oxygen is subtracted from alveolar air and carbon dioxide is added in different amounts with different kinetics [8]. Thus, ΔV_S_ estimated by pressure changes in the body plethysmograph are not only proportional to ΔV_M_ but also to ΔV_th_ and ΔV_met_.

Three strategies are available to minimize ΔV_th_ and ΔV_met_, so that ΔV_S_ ≈ ΔV_M_. The first is to ask the subject to pant, that is to breathe at a high respiratory rate with a small tidal volume (V_T_), a situation in which V_T_ remains confined in the dead space of the system [2,3,4]. The second is rebreathing in a warmed humidified bag containing carbon dioxide (~5%) [9,10,11]. These strategies minimize thermal exchange and reduce the oscillations of the composition of alveolar gasses, increasing the magnitude of ΔV_M_ relative to ΔV_th_ and ΔV_met_. The bag method is no longer used in clinical practice probably due to increased costs and to hygiene concerns. To the contrary, many commercial machines take advantage of the panting method [5,6,7]. It should be underlined, however, that panting is an artificial way of breathing, which is expected to markedly change the operating conditions of the lungs, especially in the presence of COPD [12]. The last strategy that allows to attenuate the thermal and metabolic artifacts is to subtract from ΔV_S_, electronically or digitally, a signal proportional to ΔV_th_ + ΔV_met_ (electronic or digital compensation). This task is far from simple, as warming and humidification of air in the airways during inspiration, and cooling and loss of water vapor in the box during expiration are not instantaneous processes, nor have the same kinetics [13,14], as once believed [15,16]. Indeed, in a technical note appeared in 1996, concerns were risen regarding the appropriateness of the compensation, as R_aw_, measured in healthy subjects with a plethysmograph equipped with electronic compensation, was found to increase with increasing respiratory rate faster than it could be expected on physiological ground [17]. The ability of electronic compensation to track P_alv_, and therefore R_aw_, has been questioned in infants [18]. Moreover, the exact details of the algorithm used for the compensation, at least to our knowledge, are not always reported. Despite these concerns, electronic compensation is potentially an attractive way to estimate P_alv_ during spontaneous breathing at rest, and, recently, some efforts were undertaken to investigate whether plethysmographic P_alv_ contains information that goes beyond the simple value of R_aw_ or R_aw_-related parameters, such as specific resistance or conductance [19,20], that will not be discussed in the present essay. The aim of this review is to describe how plethysmographic measurement of P_alv_ can provide additional insights in the pathophysiology of the respiratory system in patients with COPD.

## 2. Plethysmographic P_alv_-$\dot{V}$ Loops

### 2.1. Physiopathological Significance of Plethysmographic P_alv_-$\dot{V}$ Loops

Plethysmographic P_alv_ has been systematically investigated in terms of P_alv_-$\dot{V}$ plots in healthy young and elderly subjects and in COPD patients spontaneously breathing at rest [21]. A particular attention was paid to looping of the P_alv_-$\dot{V}$ relation, which was characterized by sense of rotation of the loops and area of the inspiratory (A_ins_) or expiratory loop (A_exp_).

The interest in the shape of the P_alv_-$\dot{V}$ relation lies in the fact that it reflects multiple physiopathological phenomena potentially active in the respiratory system. Indeed, in a system of rigid tubes with purely resistive characteristics, no looping should be present, and the P_alv_-$\dot{V}$ relation appears as a straight line if the flow is laminar, and a curve if the flow is turbulent. In a real respiratory system, a counterclockwise rotating loop may appear in expiration in the presence of mechanical heterogeneity, air trapping, recruitment/derecruitment of lung units, and tidal expiratory flow limitation (tEFL). During inspiration, flow-limitation is absent, and mechanical heterogeneity and air trapping produce a counterclockwise rotating loop, while recruitment/derecruitment a clockwise rotating one [11,22].

**Figure 1:**
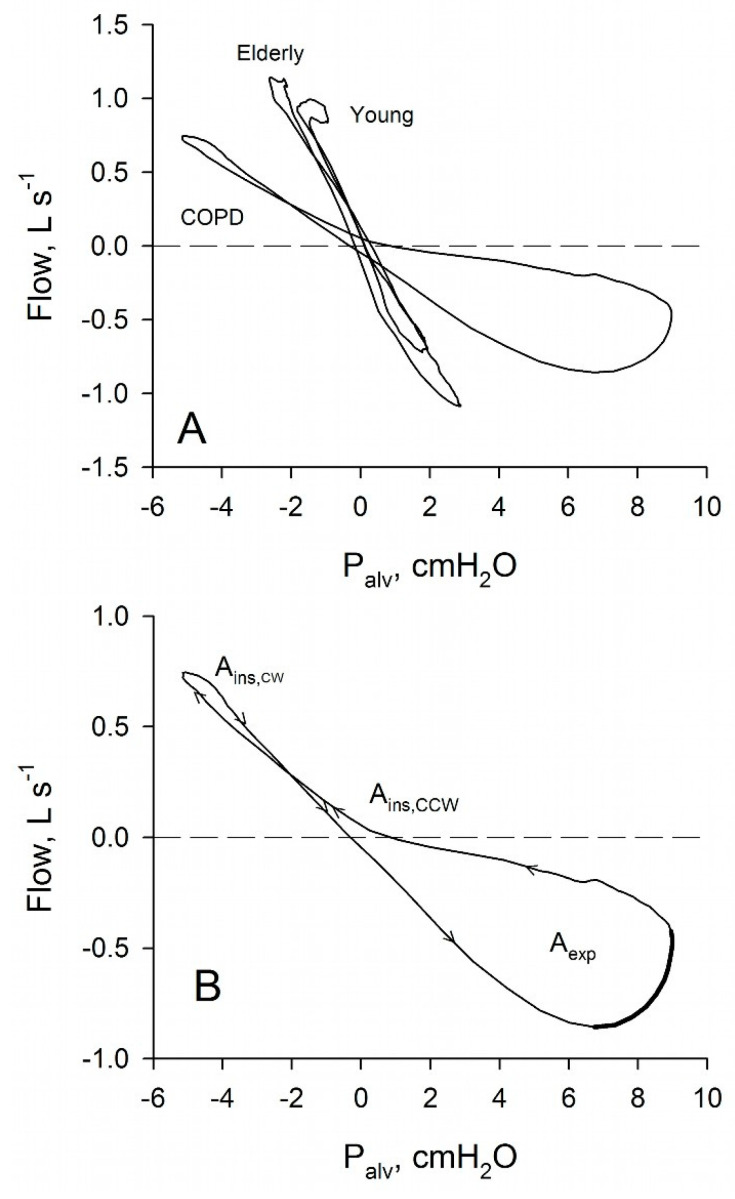
Panel (**A**): relations between P_alv_-$\dot{V}$ recorded in a young healthy subject, in an old healthy subject and in a patient with severe COPD. Panel (**B**): relation between P_alv_–$\dot{V}$ recorded in a patient with severe COPD with the indication of the sense of rotation (arrows). A_ins_: inspiratory area; A_exp_: expiratory area. The part of the expiratory P_alv_–$\dot{V}$ suggestive of the presence of expiratory flow-limitation (where flow is decreasing while driving pressure is increasing) is indicated with a thicker line. From Reference [21], with permission under the terms of the Creative Commons Attribution License.

Representative P_alv_-$\dot{V}$ plots measured in young and elderly healthy subjects and in a patient with severe COPD are shown in Figure 1. As expected, the sense of rotation was constantly counterclockwise in expiration, while it was variable in inspiration. Additionally, in all groups, A_ins_ was smaller than A_exp_, in line with the notion that, in expiration, the effects of all loop-generating factors are additive, while, in inspiration, they may cancel out. Finally, a marked increase of A_exp_ was noted in COPD patients relative to healthy subjects, as it could be expected considering the characteristics of their disease, which includes mechanical heterogeneity, recruitment/derecruitment, gas trapping, and tEFL [23,24]. In the observational study of Radovanovic et al. [21], the relative weight of the different loop-generating factors could only be hypothesized. However, several lines of evidence suggested that tEFL played a prominent role in the appearance of the large expiratory loop shown by many COPD patients. In young healthy subjects only mechanical heterogeneity can produce loops, as recruitment/derecruitment, gas trapping and tEFL are absent [23]. With aging, mechanical heterogeneity increases substantially, as indicated by the increase of the slope of phase III during the single breath nitrogen test and of the difference between static and dynamic compliance [25,26], and recruitment/derecruitment, together with gas trapping may appear [23]. Despite these changes, increases in A_exp_ during aging are small in comparison to that observed in COPD patients. Moreover, in healthy subjects, tEFL at rest is absent [23], while it is a common finding in COPD patients [24,27,28,29,30], making tEFL a good candidate to explain expiratory looping in COPD patients. Finally, in these patients, there is often a marked distortion of the expiratory loops, suggestive of an important role of tEFL in their genesis, as the other loop-generating factors tend to produce more symmetrical loops.

### 2.2. Predicting Tidal Expiratory Flow Limitation with P_alv_-$\dot{V}$ Loops

**Figure 2:**
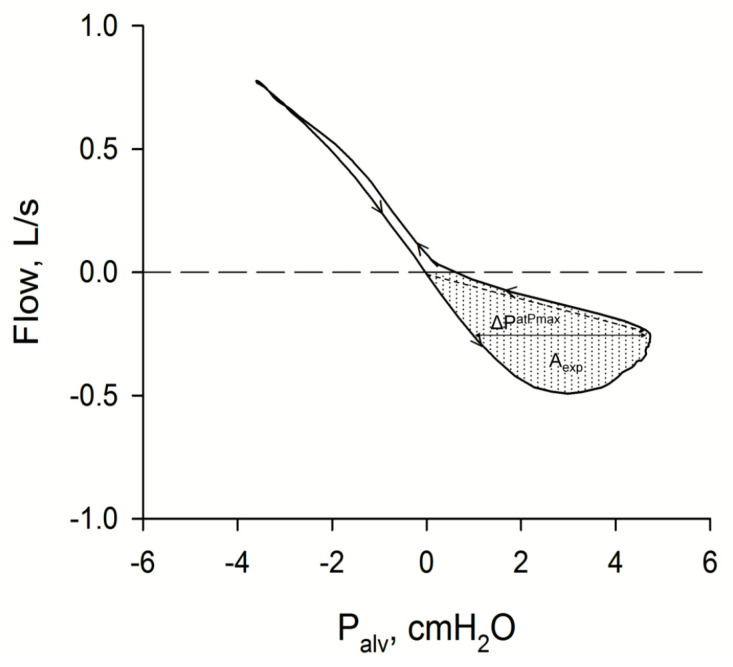
The figure shows a representative P_alv_-$\dot{V}$ plot recorded in a flow-limited COPD patients. The dotted area is the area of the expiratory loop (A_exp_). The width of the expiratory loop at maximal expiratory P_alv_, ΔP^atPmax^, is indicated by the double arrow. Small arrows indicate the sense of rotation. The slope of the broken arrow, joining the first point of expiration with the point corresponding to maximal expiratory P_alv_ is the inverse of expiratory resistance (R_exp_).

The role of tEFL in the genesis of P_alv_-$\dot{V}$ loops was specifically investigated in 60 stable COPD patients, stratified according to the absence or presence of tEFL assessed with the negative expiratory pressure (NEP) technique [30], and studied before and after bronchodilation (BD) [31]. Before BD, A_exp_ was markedly larger (360%) in patients with tEFL relative to those without tEFL. In line with previous findings [27,32,33], BD did not abolish tEFL in the majority of flow-limited patients (32/35). In these patients, the effects of BD on A_exp_ were small (−17%) relative to those of the three patients who became non-flow-limited after BD (−61%). Thus, it appears that tEFL is responsible for a large part of A_exp_ in COPD patients breathing at rest, suggesting that loop-derived parameters can be used as predictors of the presence of tEFL. This possibility was verified in the same study. In order to account for the tendency of A_exp_ to increase with increasing flow, A_exp_ was divided by peak expiratory flow, yielding a parameter, ΔP^mean^, indexing the mean width of the expiratory loop. Additionally, in an attempt to simplify the characterization of the expiratory loop, the width of the expiratory loop at maximal expiratory alveolar pressure was also calculated (ΔP^atPmax^), as shown by Figure 2. ROC analysis showed that both these indexes had an excellent ability to predict the presence of tEFL in COPD patients. Before BD, a ΔP^mean^ greater than 1.75 cmH_2_O predicted the presence of tEFL with 94.3% sensitivity and 92% specificity, and a ΔP^atPmax^ greater than 1.67 cmH_2_O, with 94% sensitivity and 96% specificity. After BD, the predictive performance of both parameters was slightly reduced, still remaining high (AUC from 0.97 to 0.90 for ΔP^mean^, and from 0.99 to 0.89 for ΔP^atPmax^). Surprisingly, expiratory resistance (R_exp_), measured as the inverse of the slope of the line joining the first point of the expiration with the point of maximal expiratory P_alv_ (Figure 2), was also a good predictor of tEFL: this is probably because, in presence of tEFL, an increase of P_alv_ does not elicit an increase in flow, causing apparent resistance to increase.

It should be underlined that the study by Pecchiari et al. [31] was not a validation study and was not powered to detect small differences in the predictive ability of the various parameters, especially when this was rather high. Nevertheless, the plethysmographic method for tEFL detection appears a novel and non-invasive tool to characterize the mechanical abnormalities of COPD patients, which does not require additional dedicated devices.

The shape of P_alv_-$\dot{V}$ plots (in the form of ΔV_M_-$\dot{V}$ plots) has also been described with different parameters by Topalovic et al. [34] in healthy subjects and in patients with asthma and COPD. No difference in the shape of P_alv_-$\dot{V}$ loops was found between healthy subjects and asthmatic patients. To the contrary, also in this study, expansion of the expiratory loop was evident in COPD patients, especially in those with a more severe disease. Interestingly, induction of bronchoconstriction in asthmatic patients resulted in small changes in the shape and extension of the expiratory loops. These data are compatible with the notion that tEFL is the major determinant of the expansion of the expiratory P_alv_-$\dot{V}$ loop as previously suggested [21,31], and it is tempting to speculate that the “openers” and “non-openers” of Topalovic et al. were patients with or without tEFL. In addition, the limited changes of the expiratory loops in asthmatics after methacholine are in line with this concept: indeed, most of stable asthmatic patients are not flow-limited, and only in a minority of them tEFL appears when methacholine is administered [35]. However, it should be recognized that caution should be taken in comparing the results of these studies, as the loops recorded by Pecchiari et al. were obtained during spontaneous breathing, while those of Topalovic et al. during paced breathing at 1 Hz.

### 2.3. Other Available Methods to Assess Tidal Expiratory Flow-Limitation

Expiratory flow-limitation is a condition in which expiratory flow at iso-volume becomes independent of the pressure difference between the mouth and the alveoli [36,37,38,39,40,41]. When this condition occurs during tidal breathing, it is referred as tidal expiratory flow limitation. Several different techniques are currently available for tEFL detection [39]. Here, they will be briefly discussed in reference to the new plethysmographic method described above.

The standard reference for tEFL detection is the negative expiratory pressure (NEP) technique, which consists of the application of a modest negative pressure at the mouth while recording the airflow and the volume. The superimposition of the flow-volume loops measured with or without NEP application can show if the increase of the driving pressure due to NEP application leads to an increase of the expiratory flow at iso-volume (tEFL absent) or not (tEFL present) [27,28,42,43,44,45]. Thus, according to the definition of tEFL, the NEP technique constitutively detects tEFL. This non-invasive technique cannot be used in patients with enhanced upper airways collapsibility, as snorers [46] and OSAH patients [47,48]. Indeed, by applying a negative pressure at the mouth, the transmural pressure at the level of the extrathoracic airways suddenly falls, and the consequent increase of upper airway resistance concomitant to the increase of the driving pressure may blunt the increase of expiratory flow (if the subject is non flow-limited) or even cause the flow to decrease relative to control. However, in subjects without enhanced upper airways collapsibility, the application of NEP up to −7 cmH_2_O does not affect the extrathoracic airways [46].

The effects of NEP can be obtained by the application of a positive pressure on the outer surface of the respiratory system. tEFL has been in fact detected through manual compression of the abdomen at rest and during exercise [49,50]. This technique does not require any special equipment, but it requires the absence of an undue abdominal muscle contraction during the maneuver.

Hyatt identified tEFL by superimposing the flow-volume loop obtained during spontaneous breathing to the one obtained during a forced expiration from total lung capacity [51]. This technique, requiring forced maneuvers, is prone to artefacts related to gas compression, unless a plethysmograph is used [52], and to the effects of previous volume and time history [53]. The latter problem is overcome by performing submaximal expiratory maneuvers instead of full forced deflation from total lung capacity [54]; however, the level of cooperation required to execute the maneuvers correctly is high, and, without an esophageal balloon, it may be difficult to check if the maneuver is performed correctly.

Other methods, like the Mead–Whittenberger and the forced oscillation technique (FOT), detect tEFL exploiting some of its secondary effects. In this sense, their assessment of tEFL is indirect, as it happens for the plethysmographic technique, which infers the presence of tEFL from the dimensions and the morphology of the expiratory P_alv_-$\dot{V}$ loops. The Mead–Whittenberger method was originally developed to separate the quasi-static and the dynamic components of pleural pressure using an esophageal balloon [55], but tEFL can supposedly inferred from an increase of dynamic pressure in front of decreasing or fixed expiratory flow [56]. On the other hand, the FOT method infers the presence of tEFL from a within-breath difference between inspiratory and expiratory reactance [56]. Despite the potential lack of specificity which may arise from the indirectness of these measurements, both the plethysmographic and the FOT methods have shown an acceptable degree of agreement with the NEP technique for the purpose of tEFL detection [31,57].

## 3. Plethysmographic Measurement of Intrinsic PEEP in Spontaneously Breathing Subjects

The coherent picture which has emerged from the studies on plethysmographic loops suggests the possibility that the P_alv_ signal recorded by electronically compensated plethysmographs is free from major artifacts due to an imperfect compensation. This may be true at least in COPD patients, in whom the increase of R_aw_ causes an increase of ΔV_M_ relative to ΔV_th_ + ΔV_met_. On this assumption, we tried to estimate intrinsic positive end-expiratory pressure (PEEPi) in stable COPD patients breathing at rest using a commercial electronically compensated body box (MasterScreen Body Plethysmograph; Erich Jaeger GmbH, Würzburg, Germany). PEEPi is a parameter related to dynamic hyperinflation, and it is responsible for a number of deleterious hemodynamic and respiratory effects [12,58].

The data that will be presented here have been collected from plethysmographic tracings recorded in our laboratories in 20 young healthy and 20 elderly healthy subjects [23], as well as in 35 flow-limited and 25 non-flow-limited stable COPD patients, before and after the administration of salbutamol [31].

**Figure 3:**
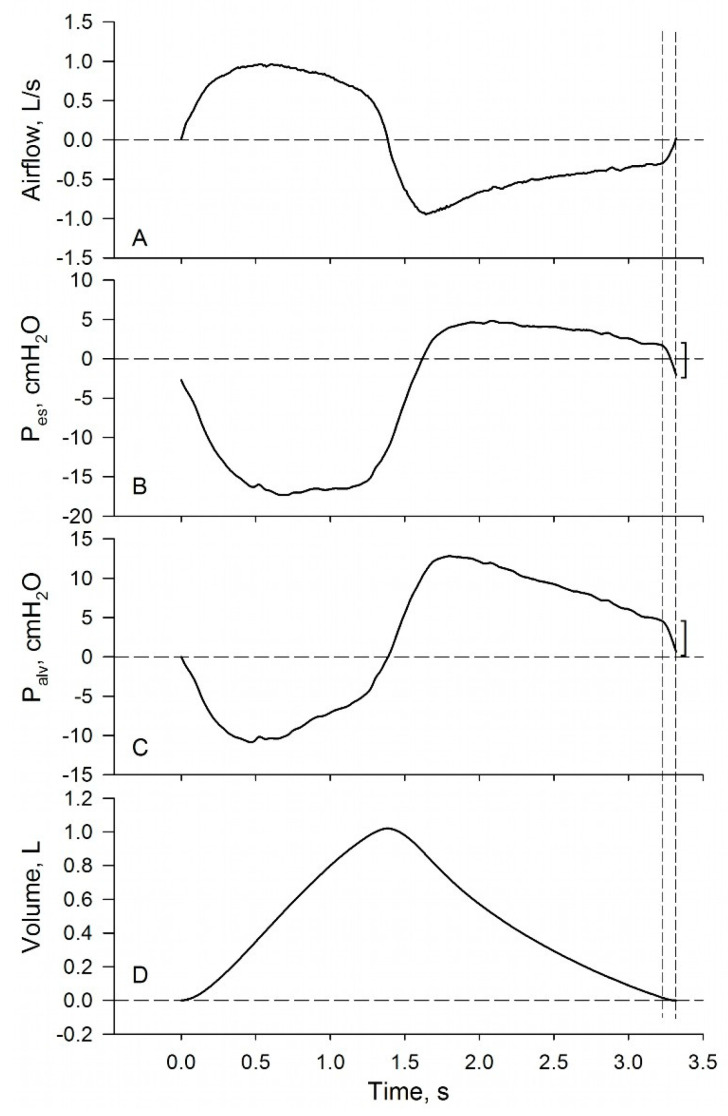
Time course of flow (panel (**A**)), esophageal pressure (P_es_) (panel (**B**)), P_es_-derived alveolar pressure (P_alv_) (panel (**C**)), and volume change (panel (**D**)) recorded during a breathing cycle in a flow-limited patient spontaneously breathing at rest. P_alv_ was calculated with the subtraction method from volume and P_es_ [55]. End-expiration is indicated by the vertical broken line on the right. The vertical broken line on the left marks the time at which P_es_ decreases rapidly, indicating, in the absence of expiratory muscles activity, decompression of alveolar air by the inspiratory muscles. The parenthesis on the P_es_ and P_alv_ tracings indicate the pressure drop corresponding to PEEPi.

In spontaneously breathing subjects, PEEPi can be estimated with esophageal manometry [59,60,61], by measuring the sudden drop of P_es_ immediately before end-expiration, as shown in Figure 3. Indeed, according to the subtraction method [55], if the viscous and viscoelastic resistances of the lung parenchyma are disregarded, ΔP_es_ ~ ΔP_alv_ when the transition between expiration and inspiration takes place so quickly that the volume change, and therefore static recoil change, is small. In turn, as long as respiratory muscles are inactive, ΔP_alv_ represents the recoil of the respiratory system which exists immediately before end-expiration (PEEPi) and is proportional to the level of hyperinflation of the subject.

We reasoned that the availability of the time course of plethysmographic P_alv_ could enable us to estimate PEEPi non-invasively without the introduction of the esophageal balloon, a requirement that has in fact limited up to now the spread of this technique in the routine clinical practice.

**Figure 4:**
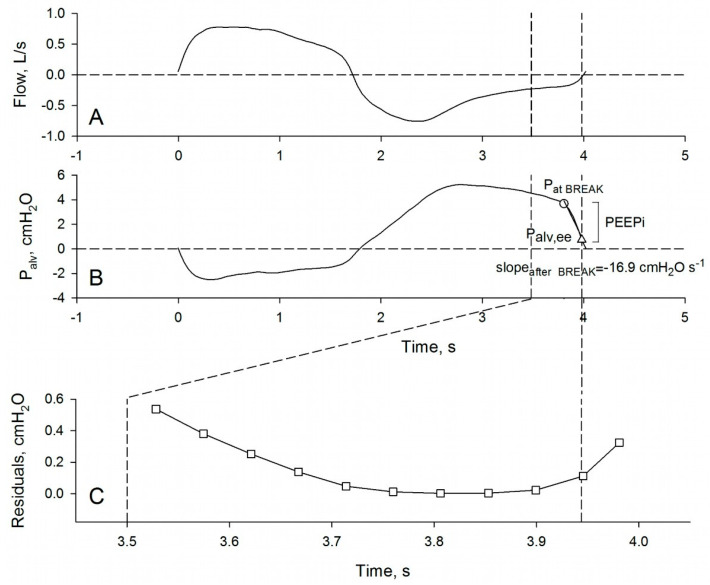
Time course of flow (panel (**A**)) and alveolar pressure (P_alv_) (panel (**B**)) in a representative severe COPD patient, breathing spontaneously at rest in the plethysmograph. The two vertical broken lines indicate the last 0.5 s of the expiration. In (**B**), the circle indicates the point of sudden pressure drop of P_alv_ (P_at BREAK_), and the triangle indicates end-expiratory P_alv_ (P_alv,ee_) (at zero flow). The slope of the P_alv_ drop after P_at BREAK_ has been obtained by interpolation of the last part of the time-P_alv_ relation (continuous straight line between circle and triangle, SLOPE_after BREAK_). Panel (**C**) shows the sum of square deviations (residuals) from the two second-order polynomials which interpolate the P_alv_ tracing in the last 0.5 s of the expiration. P_at BREAK_ is identified as P_alv_ at the minimal sum of residuals (for explanation, see the text).

On plethysmographic P_alv_ tracings, we assessed PEEPi as the difference between P_alv_ immediately before the sudden pressure drop at end-expiration (P_at BREAK_) and end-expiratory P_alv_ (P_alv,ee_), that is, P_alv_ at the point of zero flow (Figure 4). Subtraction of P_alv,ee_ from P_at BREAK_ is necessary because the former parameter is related to mechanical heterogeneity of lung parenchyma and not necessarily to hyperinflation [21,62]. Moreover, in this way, the difference between P_at BREAK_ and P_alv,ee_ (P_at BREAK_-P_alv,ee_) can be easily compared to the PEEPi measured with the esophageal balloon, as end-expiratory P_alv_ is zero by definition when assessed with the subtraction method [55].

In order to automatically measure P_at BREAK_, we applied to the last 0.5 s of expiration (region of interest, ROI) a recursive procedure aimed to identify the point of sudden slope change. Practically, P_alv_ at a certain time-point is considered P_at BREAK_, if at that time-point two second-order polynomial functions (one from the beginning of the ROI to the time-point, and one from the time-point to the end of the ROI) better fit the experimental tracing than at any other time-point. The procedure is illustrated by Figure 4 and described in detail below.

First, each time point of the last 0.5 s of the expiration was indexed from i = 0 to i = n. Second, from i = 1 to i = n − 1, the time-P_alv_ tracing was divided into two segments, from 0 to i, and from i to n, and each segment was interpolated by a second-order polynomial function. The point-to-point square deviations of the two polynomial interpolations from the measured tracing were summed, and this sum assigned to point i. P_at BREAK_ was defined as the P_alv_ at the point corresponding to the minimal sum of the square deviations (Figure 4C).

Importantly, the procedure indicated above always identifies a finite value of P_at BREAK_-P_alv,ee_ because, whatever the shape of the time-P_alv_ relation in the last 0.5 s of expiration, there will always be a time point at which the sum of square deviations is minimal. Therefore, in order for P_at BREAK_-P_alv,ee_ to be considered PEEPi, two additional criteria were introduced. First, it was required that the rate of decline of P_alv_ after P_at BREAK_ (SLOPE_after BREAK_) was adequately high, and second, that the time between the start of the fall of P_alv_ and the beginning of the next inspiration (ΔT_at BREAK_) was sufficiently small. The SLOPE_after BREAK_ was quantified as the slope of the line interpolating the time-P_alv_ relation from P_at BREAK_ to 5 points after (each point corresponding to a time interval of 0.04 ± 0.01 s) (Figure 4B). On the grounds that healthy young subjects do not show PEEPi, while flow-limited COPD patients likely do [58,59,60], the cut-off value of SLOPE_after BREAK_ was set at −10 cmH_2_O s^−1^, the value that better discriminated between healthy young and flow-limited COPD subjects in terms of receiver operating characteristics (ROC) curve. The cut-off value of ΔT_at BREAK_ (0.22 s) was chosen on the base of the few data reported by literature [58,63,64,65,66]. PEEPi was considered equal to P_at BREAK_-P_alv,ee_ if SLOPE_after BREAK_ < −10 cmH_2_O s^−1^ and ΔT_at BREAK_ < 0.22 s; otherwise, PEEPi was set to zero.

**Figure 5:**
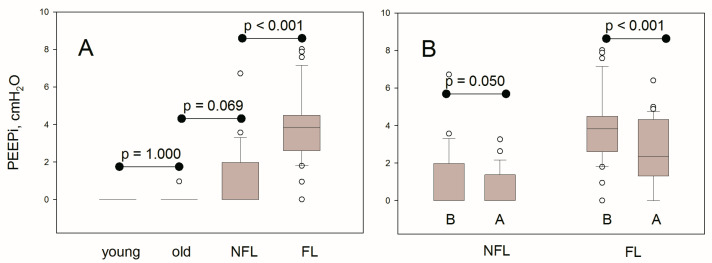
Panel (**A**) shows intrinsic positive end-expiratory pressure (PEEPi) measured in 20 young healthy subjects, 20 elderly healthy subjects, and in 60 COPD patients who were non-flow-limited (NFL, n = 25) or flow-limited (FL, n = 35) before salbutamol administration (panel (**A**)). Panel (**B**) shows the changes of PEEPi before (**B**) and after (**A**) salbutamol administration in the same COPD patients.

Table 1 and Figure 5A show alveolar pressure-derived parameters measured in healthy subjects and COPD patients before bronchodilation (values are medians (interquartile range), unless stated otherwise). P_at BREAK_, P_alv,ee_ and their difference were small or trivial in healthy subjects, increased in non-flow-limited COPD patients, but much more in flow-limited ones. According to the algorithm presented above, PEEPi was absent in all healthy subjects with the exception of one elderly subject. All but one flow-limited COPD patients and 11 out of 25 non-flow-limited patients presented PEEPi. In the 11 non-flow-limited COPD patients that exhibited PEEPi, the latter was 2.0 (1.4) cmH_2_O, significantly lower (*p* = 0.007) than that in flow-limited patients (3.8 (1.8) cmH_2_O). Considering all COPD patients together, before salbutamol, PEEPi was inversely correlated with IC %p (R_S_ −0.458, *p* < 0.001), VC %p (R_S_ −0.313, *p* < 0.001), FVC %p (R_S_ −0.444, *p* < 0.001), and FEV_1_ %p (R_S_ −0.665, *p* < 0.001), and positively correlated with RV %p (R_S_ 0.556, *p* < 0.001), ITGV %p (R_S_ 0.530, *p* < 0.001), and dyspnea at rest (R_S_ 0.518, *p* < 0.001).

Figure 5B shows the effects of salbutamol administration in non-flow-limited and flow-limited patients. Considering only COPD patients with PEEPi before salbutamol, bronchodilation significantly decreased PEEPi both in flow-limited (Δ = −1.1 (2.0) cmH_2_O, *p* < 0.001) and non-flow-limited patients (Δ =−0.9 (2.0) cmH_2_O, *p* = 0.033).

These results are very similar to those of the few studies investigating PEEPi in stable COPD patients using esophageal manometry [59,60].

Haluszka et al. studied 96 patients, finding a PEEPi of 3.0 ± 1.5 cmH_2_O (mean ± SD) in patients with FEV_1_ < 35%p, and 1.0 ± 1.5 cmH_2_O in patients with FEV_1_ ≥ 35%p [60]. As no explicit indication was given, we assume that these patients were under therapy. The post-bronchodilator values of plethysmographic PEEPi in our patients with FEV_1_ < 35%p (n = 20) or ≥35%p (n = 40) were 2.8 ± 2.0 and 1.2 ± 1.2 cmH_2_O, respectively, not significantly different from those reported by Haluszka et al. (*p* = 0.665 and 0.487, respectively). The dependencies of esophageal PEEPi reported by Haluszka et al. compared with those assessed by means of plethysmographic PEEPi after bronchodilation are shown in Table 2, and, indeed, they appear very similar.

In 10 stable COPD patients, Dal Vecchio et al. [59] measured with the esophageal balloon a decrease of PEEPi from 2.5 ± 1.5 to 0.9 ± 1.3 cmH2O (−64%) after fenoterol administration, paralleled by an increase of FEV_1_ from 1.35 ± 0.56 to 1.82 ± 0.93 L (+34%). In our study, after salbutamol PEEPi decreased and FEV_1_ increased less, from 2.6 ± 2.2 to 1.7 ± 1.8 cmH_2_O (−35%), and from 1.07 ± 0.50 to 1.14 ± 0.51 L (+7%), respectively. This apparent discrepancy is likely due to the fact that Dal Vecchio et al. used a fenoterol dose four times higher than the usual therapeutic dose, enough to induce tremors, and, consequently, the effect of bronchodilation was greater than the one that can be expected using standard doses of salbutamol, as done in our study (four 100 μg inhalations through a metered-dose inhaler and a spacer).

All together, these results suggest a good correspondence between plethysmographic and esophageal manometry-derived PEEPi. In a sense, this is surprising, considering that, in the presence of mechanical heterogeneity, alveolar pressure obtained with the two techniques can differ. Indeed, plethysmographic P_alv_ is the volume-weighted average of the individual P_alv_ of the different lung units [67], whereas P_alv_ derived from esophageal pressure is that which would exists if (a) pleural pressure was the same in all parts of the pleural space, (b) the viscoelastic properties of the lung were negligible, and (c) the lungs were homogeneous and characterized by an invariant compliance [55]. Therefore, the suggestion of an equivalence of the two techniques should be confirmed by a careful direct comparison of simultaneously recordings of plethysmographic and esophageal manometry-derived P_alv_. To our knowledge, this comparison has never been done using electronic compensation in COPD subjects, and only data relative to healthy subjects rebreathing a warmed humidified gas mixture in a volumetric plethysmograph are available [9].

Several factors can contribute to increasing the end-expiratory lung volume above the equilibrium volume of the respiratory system and, hence, to causing the presence of PEEPi. tEFL, in connection with an appropriate duration of expiration, appears to be the major cause of PEEPi [58]. However, PEEPi may also appear because of a too short expiratory duration, especially in the presence of high R_aw_, thus providing insufficient to allow the return of the respiratory system to its equilibrium volume. Moreover, in COPD subjects, expiratory narrowing of the larynx can contribute to further increase R_aw_, leading to an elevation of the end-expiratory volume [68,69,70].

Finally, it is worth it to point out a major limitation of the plethysmographic technique. Similarly to the esophageal balloon method for PEEPi estimation, at the moment, it is impossible to discriminate on P_alv_ tracings alone which part of the P_alv_ measured as PEEPi is due to the elastic recoil of the respiratory system and which is due to a change of expiratory muscles activity. Indeed, in the past, it was believed that expiratory muscles are relaxed during spontaneous breathing at rest [59,71], but, in the last few decades, a rising body of evidence indicates that, in some COPD patients, expiratory muscles are activated during expiration [72,73,74].

## 4. Conclusions

Body plethysmography is an old non-invasive technique, which, for decades, assisted clinicians in the diagnosis of respiratory diseases, providing invaluable information on non-displaceable lung volumes and airway resistance. However, recent investigations have shown that this technique is potentially able to provide additional insights in the respiratory mechanics of COPD patients, including detection of tidal expiratory flow-limitation, a further step towards a complete characterization of the mechanical abnormalities of individual COPD patients.

## Figures and Tables

**Table 1:** Alveolar pressure-derived parameters in healthy subjects and in 60 COPD patients non-flow-limited (NFL_pre_) or flow-limited (FL_pre_) before salbutamol administration.

	Young	Elderly	P(1)	NFL_pre_	P(2)	FL_pre_	P(3)
P_at BREAK_, cmH_2_O	0.46 (0.31)	0.52 (0.59)	1.000	1.21 (1.18)	0.003	4.50 (2.09)	<0.001
P_alv,ee_, cmH_2_O	0.09 (0.05)	0.10 (0.11)	1.000	0.23 (0.22)	0.003	0.76 (0.37)	<0.001
P_at BREAK-_P_alv,ee_, cmH_2_O	0.40 (0.28)	0.46 (0.52)	1.000	1.06 (1.10)	0.003	3.82 (1.80)	<0.001
PEEPi, cmH_2_O	0.0 (0.0)	0.0 (0.0)	1.000	0.0 (1.8)	0.069	3.8 (1.8)	<0.001

Values are median (interquartile range). For abbreviations, see text. P(1): probability of a difference between young and elderly healthy subjects; P(2): probability of a difference between elderly healthy subjects and non-flow-limited COPD patients; P(3): probability of a difference between COPD patients who were non-flow-limited or flow-limited before administration of salbutamol.

**Table 2:** Dependencies of esophageal PEEPi as reported by Haluszka et al. and dependencies of plethysmographic PEEPi as assessed in 60 stable COPD patients after bronchodilation in the present investigation.

	Haluszka et al.		Present Investigation	
	Esophageal PEEPi		Plethysmographic PEEPi	
	R	*p*	R	*p*
IC, % p	−0.47	<0.001	−0.36	0.005
VC, % p	−0.55	<0.001	−0.30	0.021
FEV_1_, % p	−0.56	<0.001	−0.50	<0.001
TLC, % p	−0.08	n.s.	0.26	0.047
RV, % p	0.45	<0.001	0.36	0.004
ITGV, % p	0.31	<0.01	0.39	0.002
R_aw_, cmH_2_O s L^−1^	0.69	<0.001	0.53	<0.001

IC: inspiratory capacity; VC: slow vital capacity; FEV_1_: forced expiratory volume in one second; TLC: total lung capacity; RV: residual volume; ITGV: intrathoracic gas volume; R_aw_: airway resistance. Note that airway resistance was calculated with the subtraction method in the experiments of Haluszka et al., and with the plethysmographic method in the present work. R: coefficient of correlation.

## References

[1] Dubois A.B., Botelho S.Y., Bedell G.N., Marshall R., Comroe J.H. (1956). A rapid plethysmographic method for measuring thoracic gas volume: A comparison with a nitrogen washout method for measuring functional residual capacity in normal subjects 1. J. Clin. Investig..

[2] Dubois A.B., Botelho S.Y., Comroe J.H. (1956). A new method for measuring airway resistance in man using a body plethysmograph: Values in normal subjects and in patients with respiratory disease 1. J. Clin. Investig..

[3] Briscoe W.A., Dubois A.B. (1958). The Relationship Between Airway Resistance, Airway Conductance and Lung Volume in Subjects of Different Age and Body Size. J. Clin. Investig..

[4] Butler J., Caro C.G., Alcala R., Dubois A.B. (1960). Physiological factors affecting airway resistance in normal subjects and in patients with obstructive respiratory disease. J. Clin. Investig..

[5] Coates A., Peslin R., Rodenstein D., Stocks J. (1997). Measurement of lung volumes by plethysmography. Eur. Respir. J..

[6] Goldman M., Smith H., Ulmer W. (2005). Whole-body plethysmography. Lung Function Testing.

[7] Criée C., Sorichter S., Smith H., Kardos P., Merget R., Heise D., Berdel D., Köhler D., Magnussen H., Marek W. (2011). Body plethysmography—Its principles and clinical use. Respir. Med..

[8] Sackner M.A., Feisal K.A., Dubois A.B. (1964). Determination of tissue volume and carbon dioxide dissociation slope of the lungs in man. J. Appl. Physiol..

[9] Jaeger M.J., Otis A.B. (1964). Measurement of airway resistance with a volume displacement body plethysmograph. J. Appl. Physiol..

[10] Jonson B., Bouhuys A. (1967). Measurement of alveolar pressure. J. Appl. Physiol..

[11] Matthys H. (1972). Lungenfunktionsdiagnostik Mittels Ganzkörperplethysmographie.

[12] Loring S.H., Garcia-Jacques M., Malhotra A. (2009). Pulmonary characteristics in COPD and mechanisms of increased work of breathing. J. Appl. Physiol..

[13] Peslin R., Duvivier C., Vassiliou M., Gallina C. (1995). Thermal artifacts in plethysmographic airway resistance measurements. J. Appl. Physiol..

[14] Peslin R., Duvivier C., Malvestio P., Benis A.R. (1996). Correction of thermal artifacts in plethysmographic airway resistance measurements. J. Appl. Physiol..

[15] Smidt U., Muysers K., Buchheim W., van de Woestijne K., DuBois A.B. (1969). Electronic compensation of differences in temperature and water vapour between in- and expired air and other signal handling in body plethysmography. Progress in Respiration Research, Body Plethys-Mography.

[16] Woitowitz H., Günthner W., Woitowitz R., DuBois A.B., van de Woestijne K. (1969). Some experiences with electronic simulation of BTPS conditions in body ple-thysmography. Proceedings of the International Symposium, Progress in Respiration Research, Body Plethysmography.

[17] Peslin R., Duvivier C., Malvestio P., Benis A., Polu J. (1996). Frequency dependence of specific airway resistance in a commercialized plethysmograph. Eur. Respir. J..

[18] Subbarao P., Hülskamp G., Stocks J. (2005). Limitations of electronic compensation for measuring plethysmographic airway resistance in infants. Pediatr. Pulmonol..

[19] Santus P., Radovanovic D., Henchi S., Di Marco F., Centanni S., D’Angelo E., Pecchiari M. (2014). Assessment of acute bronchodilator effects from specific airway resistance changes in stable COPD patients. Respir. Physiol. Neurobiol..

[20] Topalovic M., Derom E., Osadnik C.R., Troosters T., Decramer M., Janssens W., Belgian Pulmonary Function Study Investigators (2015). Airways resistance and specific conductance for the diagnosis of obstructive airways diseases. Respir. Res..

[21] Radovanovic D., Pecchiari M., Pirracchio F., Zilianti C., D’Angelo E., Santus P. (2018). Plethysmographic Loops: A Window on the Lung Pathophysiology of COPD Patients. Front. Physiol..

[22] Jaeger M.J., Bouhuys A. (1970). Loop Formation in Pressure vs. Flow Diagrams Obtained by Body Plethysmographic Techniques1. Clin. Exerc. Test..

[23] Pecchiari M., Radovanovic D., Santus P., D’Angelo E. (2016). Airway occlusion assessed by single breath N2 test and lung P-V curve in healthy subjects and COPD patients. Respir. Physiol. Neurobiol..

[24] Pecchiari M., Santus P., Radovanovic D., DʼAngelo E. (2017). Acute effects of long-acting bronchodilators on small airways detected in COPD patients by single-breath N2 test and lung P-V curve. J. Appl. Physiol..

[25] Buist A.S., Ross B.B. (1973). Quantitative analysis of the alveolar plateau in the diagnosis of early airway obstruction. Am. Rev. Respir. Dis..

[26] Bégin R., Renzetti A.D., Bigler A.H., Watanabe S. (1975). Flow and age dependence of airway closure and dynamic compliance. J. Appl. Physiol..

[27] Pecchiari M., Pelucchi A., D’Angelo E., Foresi A., Milic-Emili J., D’Angelo E. (2004). Effect of Heliox Breathing on Dynamic Hyperinflation in COPD Patients. Chest.

[28] D’Angelo E., Santus P., Civitillo M.F., Centanni S., Pecchiari M. (2009). Expiratory flow-limitation and heliox breathing in resting and exercising COPD patients. Respir. Physiol. Neurobiol..

[29] Eltayara L., Ghezzo H., Milic-Emili J. (2001). Orthopnea and Tidal Expiratory Flow Limitation in Patients With Stable COPD. Chest.

[30] Koulouris N.G., Valta P., Lavoie A., Corbeil C., Chassé M., Braidy J., Milic-Emili J. (1995). A simple method to detect expiratory flow limitation during spontaneous breathing. Eur. Respir. J..

[31] Pecchiari M., Radovanovic D., Zilianti C., Saderi L., Sotgiu G., D’Angelo E., Santus P. (2020). Tidal expiratory flow limitation induces expiratory looping of the alveolar pressure-flow relation in COPD patients. J. Appl. Physiol..

[32] Tantucci C., Duguet A., Similowski T., Zelter M., Derenne J.P., Milic-Emili J. (1998). Effect of salbutamol on dynamic hyperinflation in chronic obstructive pulmonary disease patients. Eur. Respir. J..

[33] Boni E., Bezzi M., Carminati L., Corda L., Grassi V., Tantucci C. (2005). Expiratory Flow Limitation Is Associated With Orthopnea and Reversed by Vasodilators and Diuretics in Left Heart Failure. Chest.

[34] Topalovic M., Exadaktylos V., Troosters T., Celis G., Aerts J.-M., Janssens W. (2017). Non-linear parameters of specific resistance loops to characterise obstructive airways diseases. Respir. Res..

[35] Tantucci C., Ellaffi M., Duguet A., Zelter M., Similowski T., Derenne J.-P., Milic-Emili J., Haworth C., Freemont A., Webb A. (1999). Dynamic hyperinflation and flow limitation during methacholine-induced bronchoconstriction in asthma. Eur. Respir. J..

[36] Pedersen O.F., Butler J.P. (2011). Expiratory Flow Limitation. Comprehensive Physiology.

[37] Tantucci C. (2013). Expiratory Flow Limitation Definition, Mechanisms, Methods, and Significance. Pulm. Med..

[38] Junhasavasdikul D., Telias I., Grieco D.L., Chen L., Gutierrez C.M., Piraino T., Brochard L. (2018). Expiratory Flow Limitation During Mechanical Ventilation. Chest.

[39] Koulouris N., Hardavella G. (2011). Physiological techniques for detecting expiratory flow limitation during tidal breathing. Eur. Respir. Rev..

[40] Wilson T.A., Rodarte J.R., Butler J.P. (2011). Wave-Speed and Viscous Flow Limitation. Comprehensive Physiology.

[41] Hyatt R.E. (2011). Forced Expiration. Comprehensive Physiology.

[42] Koulouris N.G., Dimopoulou I., Valta P., Finkelstein R., Cosio M.G., Milic-Emili J. (1997). Detection of expiratory flow limitation during exercise in COPD patients. J. Appl. Physiol..

[43] Díaz O., Villafranca C., Ghezzo H., Borzone G., Leiva A., Milic-Emili J., Lisboa C. (2001). Breathing pattern and gas exchange at peak exercise in COPD patients with and without tidal flow limitation at rest. Eur. Respir. J..

[44] Pecchiari M., Anagnostakos T., D’Angelo E., Roussos C., Nanas S., Koutsoukou A. (2009). Effect of heliox breathing on flow limitation in chronic heart failure patients. Eur. Respir. J..

[45] Koulouris N.G., Kaltsakas G., Palamidas A.F., Gennimata S.-A. (2012). Methods for Assessing Expiratory Flow Limitation during Tidal Breathing in COPD Patients. Pulm. Med..

[46] Tantucci C., Duguet A., Ferretti A., Mehiri S., Arnulf I., Zelter M., Similowski T., Derenne J.-P., Milic-Emili J. (1999). Effect of negative expiratory pressure on respiratory system flow resistance in awake snorers and nonsnorers. J. Appl. Physiol..

[47] Liistro G., Veriter C., Dury M., Aubert G., Stanescu D. (1999). Expiratory flow limitation in awake sleep-disordered breathing subjects. Eur. Respir. J..

[48] Verin E., Tardif C., Portier F., Similowski T., Pasquis P., Muir J.F. (2002). Evidence for expiratory flow limitation of extrathoracic origin in patients with obstructive sleep apnoea. Thorax.

[49] Ninane V., LeDuc D., Kafi S.A., Nasser M., Houa M., Sergysels R. (2001). Detection of Expiratory Flow Limitation by Manual Compression of the Abdominal Wall. Am. J. Respir. Crit. Care Med..

[50] Kafi S.A., Serste T., LeDuc D., Sergysels R., Ninane V. (2002). Expiratory flow limitation during exercise in COPD: Detection by manual compression of the abdominal wall. Eur. Respir. J..

[51] E Hyatt R. (1961). The interrelationships of pressure, flow, and volume during various respiratory maneuvers in normal and emphysematous subjects. Am. Rev. Respir. Dis..

[52] Ingram R.H., Schilder D.P. (1966). Effect of gas compression on pulmonary pressure, flow, and volume relationship. J. Appl. Physiol..

[53] D’Angelo E., Prandi E., Milic-Emili J. (1993). Dependence of maximal flow-volume curves on time course of preceding inspiration. J. Appl. Physiol..

[54] Pellegrino R., Brusasco V. (1997). Lung hyperinflation and flow limitation in chronic airway obstruction. Eur. Respir. J..

[55] Mead J., Whittenberger J.L. (1953). Physical Properties of Human Lungs Measured During Spontaneous Respiration. J. Appl. Physiol..

[56] Dellacà R., Santus P., Aliverti A., Stevenson N., Centanni S., Macklem P., Pedotti A., Calverley P. (2004). Detection of expiratory flow limitation in COPD using the forced oscillation technique. Eur. Respir. J..

[57] Dellacà R.L., Duffy N., Pompilio P.P., Aliverti A., Koulouris N.G., Pedotti A., Calverley P.M.A. (2006). Expiratory flow limitation detected by forced oscillation and negative expiratory pressure. Eur. Respir. J..

[58] Laghi F., Goyal A. (2011). Auto-PEEP in respiratory failure. Minerva Anestesiol.

[59] Dal Vecchio L.D., Polese G., Poggi R., Rossi A. (1990). “Intrinsic” positive end-expiratory pressure in stable patients with chronic obstructive pulmonary disease. Eur. Respir. J..

[60] Haluszka J., Chartrand D.A., Grassino A.E., Milic-Emili J. (1990). Intrinsic PEEP and Arterial P CO_2_ in Stable Patients with Chronic Obstructive Pulmonary Disease. Am. Rev. Respir. Dis..

[61] Petrof B.J., Legare M., Goldberg P., Milic-Emili J., Gottfried S.B. (1990). Continuous Positive Airway Pressure Reduces Work of Breathing and Dyspnea during Weaning from Mechanical Ventilation in Severe Chronic Obstructive Pulmonary Disease. Am. Rev. Respir. Dis..

[62] Otis A.B., McKerrow C.B., Bartlett R.A., Mead J., McIlroy M.B., Selverstone N.J., Radford E.P. (1956). Mechanical Factors in Distribution of Pulmonary Ventilation. J. Appl. Physiol..

[63] Gorini M., Villella G., Ginanni R., Augustynen A., Tozzi D., Corrado A. (2002). Effect of assist negative pressure ventilation by microprocessor based iron lung on breathing effort. Thorax.

[64] Bellani G., Coppadoro A., Patroniti N., Turella M., Marocco S.A., Grasselli G., Mauri T., Pesenti A. (2014). Clinical Assessment of Auto-positive End-expiratory Pressure by Diaphragmatic Electrical Activity during Pressure Support and Neurally Adjusted Ventilatory Assist. Anesthesiology.

[65] Appendini L. (1999). About the relevance of dynamic intrinsic PEEP (PEEPi, dyn) measurement. Intensive Care Med..

[66] Bégin P., Grassino A. (1991). Inspiratory Muscle Dysfunction and Chronic Hypercapnia in Chronic Obstructive Pulmonary Disease. Am. Rev. Respir. Dis..

[67] Peslin R. (1968). Theoretical analysis of airway resistances on an inhomogeneous lung. J. Appl. Physiol..

[68] Campbell A.H., Imberger H., Jones B.M. (1976). Increased upper airway resistance in patients with airway narrowing. Br. J. Dis. Chest.

[69] Baz M., Haji G.S., Menzies-Gow A., Tanner R.J., Hopkinson N.S., Polkey M.I., Hull J.H. (2015). Dynamic laryngeal narrowing during exercise: A mechanism for generating intrinsic PEEP in COPD?. Thorax.

[70] Higenbottam T., Payne J. (1982). Glottis narrowing in lung disease. Am. Rev. Respir. Dis..

[71] Campbell E., Friend J. (1955). Action of breathing exercises in pulmonary emphysema. Lancet.

[72] Ninane V., Rypens F., Yernault J.-C., De Troyer A. (1992). Abdominal Muscle Use during Breathing in Patients with Chronic Airflow Obstruction. Am. Rev. Respir. Dis..

[73] Ninane V., Yernault J.-C., De Troyer A. (1993). Intrinsic PEEP in Patients with Chronic Obstructive Pulmonary Disease: Role of Expiratory Muscles. Am. Rev. Respir. Dis..

[74] Zakynthinos S.G., Vassilakopoulos T., Zakynthinos E., Roussos C. (1997). Accurate measurement of intrinsic positive end-expiratory pressure: How to detect and correct for expiratory muscle activity. Eur. Respir. J..

